# Effect of Glycosaminoglycan Replacement on Markers of Interstitial Cystitis *In Vitro*

**DOI:** 10.3389/fphar.2020.575043

**Published:** 2020-12-03

**Authors:** Peadar Rooney, Christina Ryan, Barry J. McDermott, Kapil Dev, Abhay Pandit, Leo R. Quinlan

**Affiliations:** ^1^ CÚRAM SFI Research Centre for Medical Devices, National University of Ireland Galway, Galway, Ireland; ^2^ Physiology, School of Medicine, National University of Ireland Galway, Galway, Ireland; ^3^ Translational Medical Device Lab, National University of Ireland Galway, Galway, Ireland

**Keywords:** biomaterial, inflammation, barrier function, interstitial cystitis, glycosaminoglycan

## Abstract

**Aims:** To examine the effect of three commercial intravesical formulations of glycosaminoglycan on *in vitro* inflammatory models of IC/BPS to better understand there effect on specific markers of disease.

**Methods:** Human urothelial cells (HTB-4) were cultured under four conditions in the presence or absence of commercial GAG formulations. Cells were cultured under a basal condition or pre-treated with protamine sulfate (100 ng/ml) (damages the endogenous glycosaminoglycan layer), hydrogen peroxide (1%) (a metabolic stressor) or TNFα (10 ng/ml) (creating an inflammatory environment). Each of these four culture conditions was then treated with one of three GAG formulations, CystistatⓇ, iAluRilⓇ and HyacystⓇ. Assays were then performed to examine the effect of the exogenous GAGs on cell viability, cell migration, sGAG production, cytokine and gene expression.

**Results:** All GAG formulations were well tolerated by the HTB-4 cells and supported cell growth and migration. iAluRilⓇ was most effective at stimulating endogenous sGAG production under all conditions, increasing sGAGs by up to 15-fold. All GAG formulations significantly reduced the production of the pro-inflammatory cytokine IL-8 under basal conditions, while no GAG treatment suppressed cytokine production under any other condition. Only Cystistat^Ⓡ^ had a significant effect on HA receptor expression, significantly increasing ICAM-1 expression at 3 h that returned to basal levels at 24 h. No GAG treatment significantly changed the expression of GAG synthesis enzymes (CSGALNACT1, CSGALNACT2) or markers of tissue remodeling (MMP2, TIMP1) and pain (COX-1/PTGS-1, NGF).

**Conclusions:** The data presented in this study reveal that commercial intravesical formulation support cell viability and migration. In addition, the commercial GAG formulations have a mild anti-inflammatory effect in the *in vitro* model of interstitial cystitis/bladder pain syndrome.

## Introduction

Interstitial cystitis/bladder pain syndrome (IC/BPS) is a chronic bladder condition, for which the criteria for diagnosis was originally developed by the National Institute of Diabetes and Digestive and Kidney disease (NIDDK) (Gillenwater and Wein, 1988; Wein et al., 1990) which was subsequently updated the European Society for the Study of Interstitial Cystitis (ESSIC) (Van de Merwe et al., 2008). The current ESSIC definition of BPS is a chronic condition (greater than 6 months) with pelvic pain, pressure, or discomfort perceived to be related to the urinary bladder, accompanied by at least one other urinary symptom such as persistent urge to void or frequency in the absence of an identifiable cause (Van de Merwe et al., 2008). Literature reports two types of IC/BPS, the classical form and a non-ulcerated form of the condition. The global prevalence of IC/BPS is debated with reports varying from 0 one to five per 1,000 population (Leppilahti et al., 2002; Clemens et al., 2005; Dinis et al., 2015; Lee et al., 2018). The health care costs associated with managing IC/BPS are significant, and the impact on patient quality of life can be very severe (Chancellor, 2007; Hakimi et al., 2017). The impact of IC/BPS can be so significant that in certain cases patients report lower quality of life scores than patients with end-stage renal disease who are receiving dialysis (Nickel et al., 2010; Sutcliffe et al., 2015).

The etiology of IC/BPS remains unclear, despite a significant research effort over many years and numerous hypotheses have been suggested. It is clear is that IC/BPS is a multifactorial condition involving both genetic and environmental factors. A significant body of work reports that alterations affecting the urothelium as a key component in IC/BPS. In the normal healthy bladder the urothelium forms the luminal interface with the urinary solutes generating a functionally tight urothelial barrier that restricts the movement of solutes into the underlying tissues (Montalbetti et al., 2017). Damage to the urothelial layer leads to leakiness and increased permeability, allowing solutes permeate into the sub-urothelium, which is a key factor in disease pathophysiology (Chai et al., 2016). The barrier function of the urothelium is associated with a thick mucus coating consisting of a variety of proteoglycans, composed of a protein core and negatively charged glycosaminoglycan (GAG) side chains (Madersbacher et al., 2013; Rooney et al., 2015). This so-called GAG layer is the major component of the luminal extracellular matrix of the apical bladder surface lining (Cervigni, 2015). As the GAG components are typically hydrophilic, they create a hydrating layer luminal to the urothelium and contribute to barrier formation, which prevents attachment by bacteria, and leakage of proteins, urinary solutes and ions into the urothelial sub-layers (Cervigni, 2015).

As the cause of IC/BPS is still unknown, it has proved difficult to develop a definitive treatment regimen, thus there exists a wide variety of therapies often reporting conflicting results. However, there is now a general agreement that alternations in, or loss of, the GAG layer is an important component in the IC/BPS etiology and progression (Lazzeri et al., 2016). There are a range of commercial treatment approaches available to patients. These options including disease specific education programs, behavioral modification through diet management, chemical treatment, targeted therapy through oral or intravesical routes, and surgery (Tirumuru et al., 2010). Of these options an increasingly common clinical approach is employing a biomaterials approach. In this context GAG layer replacement or restoration therapies are increasing as these have been shown to reduce inflammation and can be delivered with relative ease in the clinic (Lee et al., 2015; Wyndaele et al., 2018). A number of GAG-based intravesical biomaterial based device treatments are currently available and come in the form of formulations containing hyaluronic acid (HA) alone, chondroitin sulfate (CS) alone or a combination of HA and CS, across a range of concentrations and ratios (Madersbacher et al., 2013).

While many theories have been postulated there is now some degree of certainty that inflammation plays a key role in the pathophysiology of IC/BPS (Liu et al., 2015; Maeda et al., 2015). Persistent cycles of inflammation and recurrent injury to the luminal bladder including damage to the GAG layer are key to disease pathology and highlight targets for intervention (Grover et al., 2011). Despite intravesical GAG replenishment therapies being in use for more than 15 years, evidence of their molecular mechanism of action is missing or poorly described at a cellular level and the evidence from clinical studies is fragmented (Zhang et al., 2017). There is growing but conflicting data supporting the effectiveness of intravesical GAG therapies with Engelhardt *et al.* showing a 50% remission in symptoms after intravesical HA treatment (Engelhardt et al., 2011). While Hanno *et al.* reached a different conclusion through a double-blind, placebo-controlled trial, where they did not find any significant benefit of HA compared with placebo (Hanno et al., 2011). Here we chemical insults to mimic aspects of the inflammatory components of IC/BPS *in vitro*, and we compare the effects of three commercially available GAG biomaterial medical device formulations on cellular and molecular indicators of disease. These investigations allow a better understanding of the pathology and may reveal targets for future treatments.

## Materials and Methods

All general materials were purchased from Sigma-Aldrich United States unless specifically stated otherwise. Commercial GAG formulations where chosen based on what was available in the market at the time of study. GAG formulations used in the study (CystistatⓇ (0.8 mg/ml HA), iAluRilⓇ (16 mg/ml HA, 20 mg/ml CS) or HyacystⓇ (0.8 mg/ml HA)) were all acquired directly from the respective suppliers.

### Cell Culture

Human urothelial cells HTB-4 (ATCC, Manassas, VA) were cultured in Dulbecco’s Modified Eagle’s Medium (DMEM) supplemented with 10% fetal calf serum (FCS) and 1% penicillin/streptomycin. In all cases cells were grown to 70–80% confluency and washed three times with Hanks’ Balanced Salt Solution (HBSS) before all experiments. For all experiments, cells were seeded at 50,000 cells/ml and grown for 24 h in standard media before treatments began. All experiments followed a similar timeline unless stated otherwise. Cells were challenged with the chemical insult for 1 h. The different chemical insults were used to replicate elements of IC/BPS *in vitro*. These chemical insults included:

• Protamine sulfate: Protamine sulfate was used at 100 ng/ml and has previously been used to generate an acute model of IC/BPS *in vivo*, damaging the urothelial GAG layer, reducing barrier function and is proinflammatory (Kyker et al., 2005; Lavelle et al., 2002)
• hydrogen peroxide: A 1% H2O2 solution was used as a general agent to induce cell stress
• Tumor Necrosis Factor Alpha (TNFα): To mimic a more classical proinflammatory environment, and based on previous studies (Rooney et al., 2015), cells were treated with 10 ng/ml TNFα (PreproTech, London, United Kingdom) and sampled at 3, 5, 12, and 24 h.

Following exposure to the chemical insult, the medium was replaced with fresh medium in a 1:1 ratio with one of the individual GAG formulations for 2 h. After GAG treatment the culture was washed three times in fresh medium to remove any exogenous GAG and the wash finally replaced with fresh medium and sampled at 24 h unless stated otherwise.

In all cases cell culture supernatants were harvested and stored at −20°C prior to further analysis.

### Effect of Glycosaminoglycan Treatment on Cell Viability and Cell Growth/Migration

Cell viability was measured by alamar blue assay of mitochondrial activity. The basis of this assay is a measure of the ability of the cells to reduce resazurin to resorufin. In addition, as a measure of cell growth and cell migration a wound healing assay was carried out in 48-well plates using a 200 µl pipette tip to generate a single scratch wound in the cell monolayer across the diameter of the well at time zero. At the end of the incubation period cells were fixed with 3.5% paraformaldehyde for 10 min, stained with 0.1% crystal violet (Sigma–Aldrich, Arklow, Ireland) in phosphate buffered saline (PBS) for 10 min and washed in PBS for 5 min, three times. Images of the scratch were analyzed to determine scratch wound area, using the MRI wound healing tool in FIJI from NIH.

### Effect of Glycosaminoglycan Treatment on sGAG Production and Cytokine Production

sGAG production in all cases was measured from the culture supernatants, using the Blyscan™ glycosaminoglycan assay (Biocolor, United Kingdom), performed as per manufacturer’s instructions. Cytokine production was measured by ELISA, analyzing urothelial cell supernatants. IL-6 and IL-8 Human ReadySetGo ELISAs (eBioscience, Hatfield, United Kingdom) were performed as per manufacturer’s instructions. IL-6 standards ranged from 2 to 200 pg/ml with a sensitivity of 2 pg/ml. IL-8 standards ranged from 2 to 250 pg/ml with a sensitivity of 2 pg/ml.

### Effect of Glycosaminoglycan Treatment on Gene Expression

The expression of genes (Table 1) related to the HA receptor family (CD44, ICAM, and HMMR/RHAMM), tight junction protein (ZO1), GAG synthesis enzymes (CSGALNACT1 and CSGALNACT2), markers of pain and inflammation (COX1, NGF) and tissue remodeling (TIMP1 and MMP1) were assayed. Total RNA was isolated using RNeasy Mini Kit (Qiagen, Crawley, United Kingdom) and quantity assessed by nanodrop (ND-2000 Spectrophotometer (ThermoFisher Scientific, United Kingdom). The integrity of RNA samples was assessed using a bioanalyser (Agilent, Dublin, Ireland). Samples with a 260:280 nm ratio of 1.8–2.1 for purity and an RNA integrity number greater than 8.5 were used in subsequent experiments. Isolated RNA was stored at −80 °C before transformation into cDNA using the cDNA synthesis first strand synthesis kit (Roche Life Sciences, Ireland) all cDNA stored at −20 °C. All reactions and controls were performed in triplicate using real time ready custom 384-well plates on a Lightcycler 480 system (Roche Life Sciences, Ireland). Thermal cycling conditions were as recommended by the manufacturer (Applied Biosystems, Paisley, United Kingdom). Relative changes in gene expression were determined using the ΔΔ^CT^ method.

**TABLE 1:** Gene primer sequences for qPCR.

Gene	Forward primer	Reverse primer
CD44	ccagaaggaacagtggtttggc	actgtcctctgggcttggtgtt
ICAM1	agcggctgacgtgtgcagtaat	tctgagacctctggcttcgtca
HMMR/RHAMM	ggctgggaaaaatgcagaggatg	cctttagtgctgacttggtctgc
CSGALNACT1	ggatgacgtgtcagtatcggtc	ccgtaccactatgaggttgctg
CSGALNACT2	tcatctcacagtggtgtattttgg	gcacccacatttagtcctcgt
MMP2	agcgagtggatgccgcctttaa	cattccaggcatctgcgatgag
COX1-PTGS1	gatgagcagcttttccagacgac	aactggacaccgaacagcagct
TJP1/ZO1	gtccagaatctcggaaaagtgcc	ctttcagcgcaccataccaacc
NGF	cctcatccctgtctattgctcc	gttggctccttgcttgttctgc
TIMP1	ggagagtgtctgcggatacttc	gcaggtagtgatgtgcaagagt

### Statistical Analysis

All treatments were carried out in triplicate for each experimental block and each experimental block was run on three separate occasions. All data were normalized to within block controls and comparisons were analyzed by one-way ANOVA with Dunnett’s post-test. Comparisons were determined by Tukey post hoc test to compare groups. Data are presented as the mean ± sem with statistical significance indicated as *p < 0.05.

## Results

### Effect of Glycosaminoglycan Treatment on Cell Viability

**FIGURE 1:**
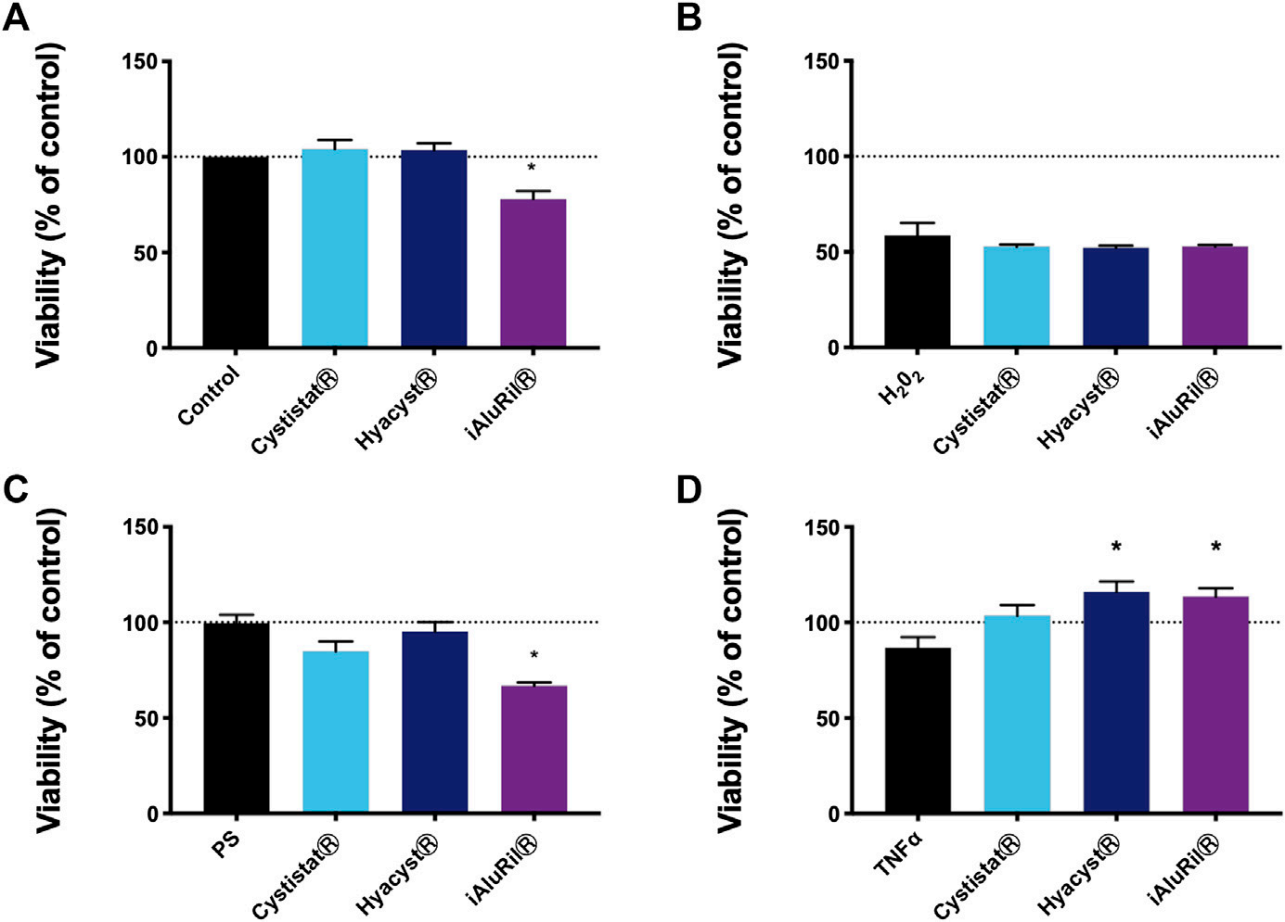
Effect of commercial GAG formulations on cellular metabolic activity under different conditions. **(A)** GAG treatment alone. **(B)** Pre-treatment with hydrogen peroxide. **(C)** Pre-treatment with protamine sulfate. **(D)** Pre-treatment with TNFα over time. All data are normalized to controls in each case where 100% is the control indicated by the horizontal line. All data are expressed as mean ± sem, *n* = 3.

Under basal, conditions neither CystistatⓇ nor HyacystⓇ had any effect on the viability of HTB-4 cells, while iAluRilⓇ reduced viability by approximately 20% (*p* < 0.011) (Figure 1A). The cell viability in general was reduced by approximately 50% in the presence of H_2_O_2_ (Figure 1B), with no significant effect seen with any of the GAG treatments. Protamine sulfate alone or pre-treatment with protamine sulfate and subsequent treatment with CystistatⓇ and HyacystⓇ had no effect of on cell viability. While similar to iAluRilⓇ alone pre-treatment with protamine sulfate and subsequent iAluRilⓇ treated cultures, viability was reduced by approximately 20% (*p* < 0.0026) (Figure 1C). In the presence of TNFα alone cell viability was not significantly reduced. However, treatment with both HyacystⓇ and iAluRilⓇ significantly increased cell viability vs. TNFα alone (approximately 30% increase, *p* < 0.017 and *p* < 0.27 respectively) (Figure 1D), while neither HyacystⓇ or iAluRilⓇ were different from CystistatⓇ.

### Effect of Glycosaminoglycan Treatment on Wound Healing

**FIGURE 2:**
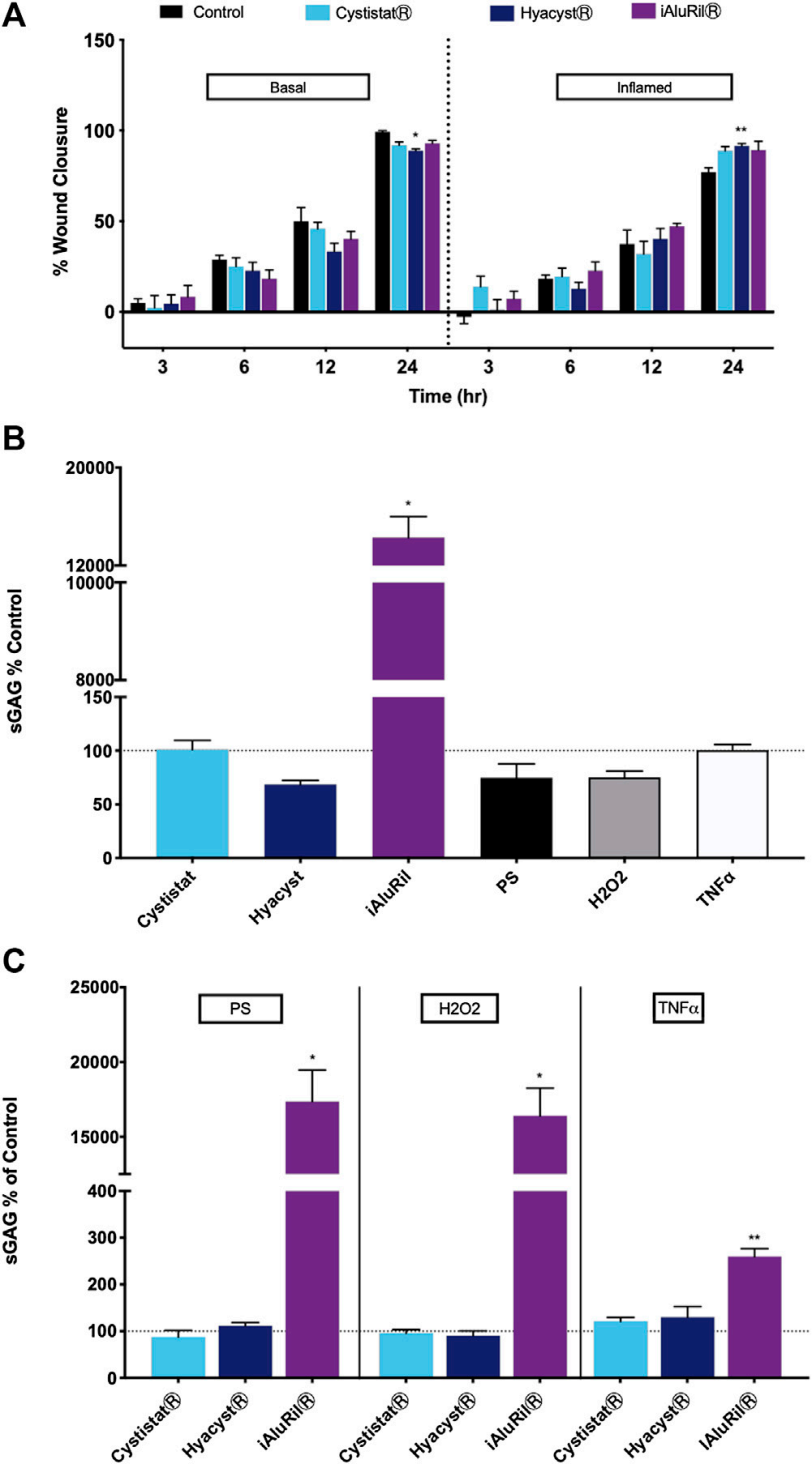
Effect of commercial GAG formulations on wound healing and sGAG production. **(A)** effect of commercial GAG formulations on wound healing in a scratch wound assay over time. *p < 0.001 V control, **p < 0.005 V control. **(B)** Effect of GAG formulations and chemical treatment on sGAG relative to control (no treatment condition). **(C)** Effect of pre-treatment with PS, H_2_O_2_ or TNFα and GAG formulations on sGAG production relative to GAG treatment alone. All data are expressed as mean ± sem, *n* = 3, *p < 0/001, **p < 0.008.

The use of the proinflammatory cytokine TNFα is commonplace in trying to replicate the *in vivo* inflammatory milieu (Hirose et al., 2016; Zhang et al., 2016). Here the effect of TNFα on wound closure was examined. After 24 h in the scratch wound assay all wounds were closed to approximately 100% in both basal and inflamed (10 ng/ml TNFα) conditions (Figure 2A). There was no significant effect of GAG formulation at 3, 6, or 12 h. At 24 h, only HyacystⓇ (*p* < 0.0001) was significantly reduced in terms of wound recovery under basal conditions. While conversely under inflammatory conditions only HyacystⓇ (*p* < 0.005) significantly improved wound recovery.

### Effect of Glycosaminoglycan Treatment on sGAG Production

Under basal conditions chemical treatment with PS, H_2_O_2_ or TNFα alone had no significant effect on sGAG production. Of the GAG formulations only iAluRilⓇ significantly affected sGAG production relative to control (Figure 2A), increasing measured sGAGs by a factor of 15 (*p* < 0.001). Pre-treatment with PS, H_2_O_2_ or TNFα resulted in no difference in sGAG production vs. GAG treatment alone except for iAluRilⓇ (Figure 2B). iAluRilⓇ significantly increased sGAG production in all cases by a factor of 15 in the case of PS and H_2_O_2_ pre-treatment (*p* < 0.001, Figure 2B). Under inflammatory conditions with TNFα all GAG treatments increased sGAG relative to GAG treatment alone. However only iAluRilⓇ significantly increased sGAG production under these conditions by a factor of three relative to control (*p* < 0.008, Figure 2B).

### Effect of Glycosaminoglycan Treatment on Cytokine Production

**FIGURE 3:**
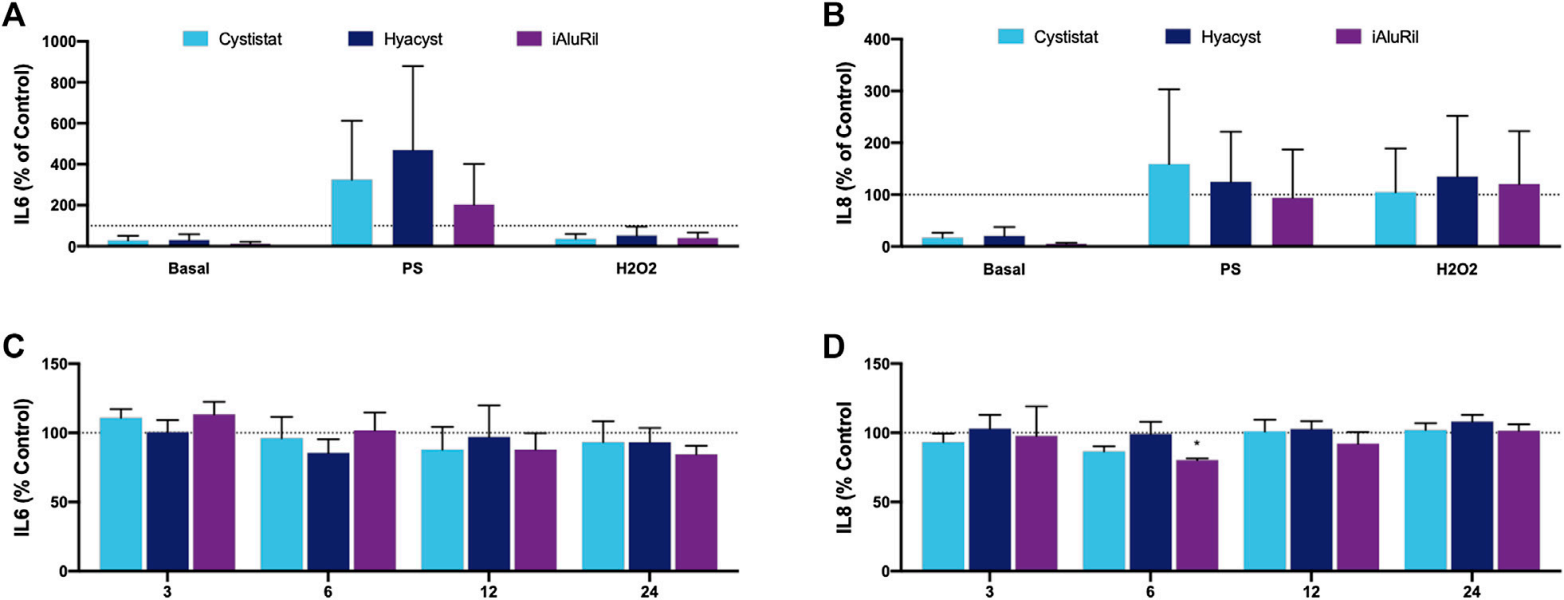
Effect of commercial GAG formulations on cytokine production as assessed by ELISA. **(A)** IL-6 production relative to control (100%). **(B)** IL-8 production relative to control (100%). **(C)** IL-6 production relative to control when pre-treatment with TNFα **(D)** IL-8 production relative to control when pre-treatment with TNFα. All data are normalized to controls for each treatment group and expressed as mean ± sem, *n* = 3.

Under basal conditions, no GAG treatment had a significant effect on IL-6 (Figure 3A), while all GAG treatments reduced IL-8 production over the 24-h treatment period (Figure 3B, *p* < 0.002). In cultures pre-treated with PS or H_2_O_2_ no GAG formulation had a significant effect on IL-6 or IL-8 production. We were interested in the potential acute (3–6 h) and delayed (12–24 h) effects of TNFα pre-treatment. Over the time course no GAG treatment significantly reduced IL-6 production (Figure 3C). iAluRilⓇ reduced IL-8 production under TNFα inflamed conditions but only at the 6-h time point (Figure 3D, *p* < 0.006).

### Effect of Glycosaminoglycan Treatment on Gene Expression

**FIGURE 4:**
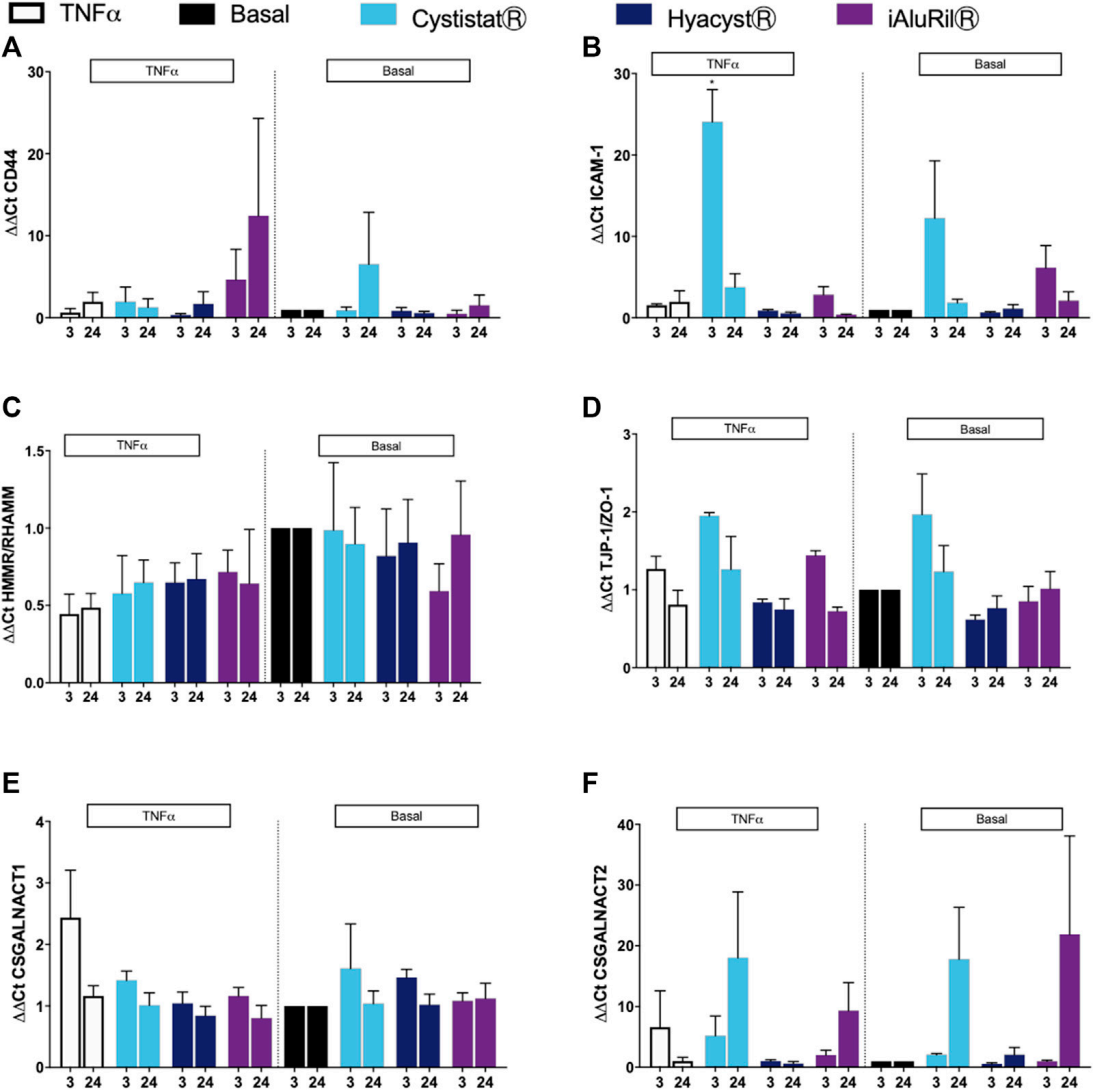
Effect of commercial GAG formulations on the expression of hyaluronic acid receptors, tight junction proteins and GAG synthesis enzymes assessed by qPCR gene array. **(A)** Effect of GAG treatment on CD44 expression under basal and TNFα conditions. **(B)** Effect of GAG treatment on ICAM expression under basal and TNFα conditions. **(C)** Effect of GAG treatment on HMMR/RHAMM expression under basal and TNFα conditions. **(D)** Effect of GAG treatment on TJP-1/ZO-1 expression under basal and TNFα conditions. **(E)** Effect of GAG treatment on CSGALNACT1 expression under basal and TNFα conditions. **(F)** Effect of GAG treatment on CSGALNACT2 expression under basal and TNFα conditions. All data are expressed as mean ± sem, *n* = 3.

TNFα represents a commonly cited model of bladder inflammation studies and is a current target for IC/BPS therapy (Bosch, 2014; Bosch, 2018; Croft et al., 2013). We examined the effect of the three commercial GAG formulations on HA family receptors and markers of barrier function under basal and TNFα conditions. There was no significant change in the expression of CD44 gene expression under basal or inflamed conditions (Figure 4A). At both 3 and 24-h under inflamed conditions cells treated with iAluRilⓇ showed increased expression in CD44, but due to high variability in the signal this did not reach significance. The HA receptor intracellular adhesion molecule-1 (ICAM-1), under basal and inflamed conditions treated with CystistatⓇ showed a significant increase in expression at 3 h (*p* < 0.048), which returned to basal levels at 24 h (Figure 4B). There was no significant effect under any condition or treatment for HA receptor HMMR/RHAMM (Figure 4C) or the tight junction protein TJP-1/ZO-1 (Figure 4D). There is the potential that GAG treatment may activate endogenous GAG production, thus we examined the expression of enzymes related to GAG synthesis including Chondroitin sulfate N-acetylgalactosaminyltransferase 1 (CSGALNACT1) and Chondroitin sulfate N-acetylgalactosaminyltransferase 2 (CSGALNACT2), both critical enzymes in the production of sGAGs. The expression of CSGALNACT1 was not altered in basal or under inflamed conditions (Figure 4E). The expression of CSGALNACT2 under both basal and inflamed conditions trends to increase at 24 h (Figure 4F), however, this was not significant due to high variability in the signal.

**FIGURE 5:**
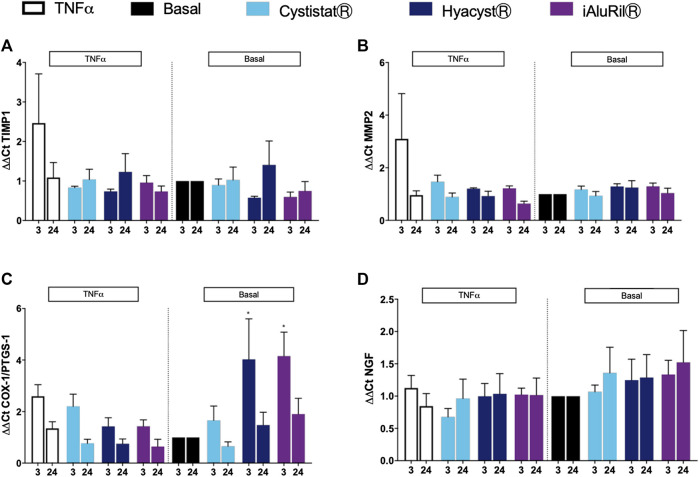
Effect of commercial GAG formulations on the expression of GAG synthesis enzymes assessed by qPCR gene array. **(A)** Effect of GAG treatment on TIMP1 expression under basal and TNFα conditions. **(B)** Effect of GAG treatment on MMP2 expression under basal and TNFα conditions. **(C)** Effect of GAG treatment on COX-1/PTGS-1 expression under basal and TNFα conditions. **(D)** Effect of GAG treatment on NGF expression under basal and TNFα conditions. All data are expressed as mean ± sem, *n* = 3.

The GAG formulations did not alter expression of genes associated with tissue remodeling TIMP1 and MMP2 under basal or inflamed conditions (Figures 5A,B). For markers of pain, HyacystⓇ and iAluRilⓇ treatment of cells under basal conditions resulted in higher expression of COX-1/PTGS at 3 h compared to control (Figure 5C), a profile that while reduced in magnitude is similar in profile at 24 h. Under inflamed conditions, both HyacystⓇ and iAluRilⓇ reduced COX-1/PTGS expression at 3 h, there was no effect on COX-1/PTGS expression at 24 h (Figure 5C). Nerve growth factor is considered a potential biomarker for IC/BPS thus we examined its expression. Under basal and inflamed conditions it can be seen that none of the GAG formulations resulted in a significant change in NGF gene expression (Figure 5D).

## Discussion

The anatomy of the urinary tract provides a simple and accessible route to targeting therapy directly to the luminal lining of the bladder wall (Kaufman et al., 2010). Intravesical treatment options for IC/BPS offer a number of advantages over oral administration including the ability to administer high concentrations of therapeutic agent directly to the affected site in the bladder, and a lower likelihood of systemic side effects (Kaufman et al., 2010). There is very limited *in vitro* data comparing the various treatment options available (Gülpınar et al., 2018; Stellavato et al., 2019a). Comparative studies at the cellular and molecular level can lead to better understanding of the pathology and may help identify promising targets for future treatments. The ideal therapeutic should be well toleration by the urothelium and promote restoration of the GAG layer and barrier function. In addition, it should be anti-inflammatory and supress nociceptive pathways.

In this study, a number of assays were performed in order to compare and examine the effect of three different GAG treatments (CystistatⓇ, iAluRilⓇ, and HyacystⓇ) on three different *in vitro* inflammatory models of IC/PBS. These *in vitro* models were based on HTB-4 cells exposed to one of three inflammatory insults: PS, H_2_O_2_ or TNFα. We examined the effect of the GAG formulations on cell viability and showed that when HTB-4 cell cultures were challenged with PS or H_2_O_2_ there was no effect of exogenous GAG. The exception was a small reduction in cell viability of approximately 20% in the PS condition with iAluRilⓇ. A number of studies have explored the effects of exogenous GAGs both HA and CS alone or in combination. For example, exogenous GAGs reduce the production and activity of proinflammatory mediators and matrix metalloproteinases in chondrocytes under inflamed conditions. In a recent *in vitro* study under a bladder model system, TNFα was used to mimic IC/BPS damage in the bladder (Stellavato et al., 2019a). In this study both exogenous HA, CS, and combination were anti-inflammatory. A similar trend was observed in the IL-6 levels in a mouse model treated with single dose HA after induction chemical cystitis (Sahiner et al., 2018). A major confounding factor is the exact molecular weight of the formulations employed as many of the physiological effects of exogenous HA are dependent on molecular weight (Hascall et al., 2004; Medina et al., 2006).

In the current study in the presence of the inflammatory cytokine TNFα both HyacystⓇ and iAluRilⓇ resulted in an increase in cell viability by approximately by 15% compared to TNFα alone (Figure 1D). The effectiveness of GAG replacement therapy as currently employed may reside in its potential to promote tissue remodeling and wound/ulcer healing. All three GAG formulations tested here contain HA with one containing a combination of HA and CS (iAluRilⓇ). The literature is rich in evidence that HA is active throughout the wound healing cycle (Chen and Abatangelo, 1999; Almond, 2007; Prosdocimi and Bevilacqua, 2012), while there is also some evidence for a role for CS in wound healing (Im et al., 2013). In this study we found no major additional benefit of GAG formulations for wound healing under basal conditions. HyacystⓇ improved wound healing significantly under inflamed conditions the magnitude (approx. 15%) of the difference was very small and unlikely to be of clinical significance. HA is known to drive different effects associated with differences in molecular weight (David‐Raoudi et al., 2008). A number of other studies have also examined the effect of exogenous HA in wound healing in a number of *in vitro* model systems (D’Agostino et al., 2015; Stellavato et al., 2019b). Interestingly very commonly there is a reported increase in low molecular weight HAs resulting in cytokine production driving angiogenesis and repair (Maneiro et al., 2004; Stern et al., 2006). This might suggest that GAGs that are more pro-inflammatory might promote more wound repair in the first instance, and explain the slightly more reparative effect of the more pro-inflammatory HyacystⓇ formulation.

As GAGs are considered critical components of the extracellular matrix and play an important role in barrier function in the bladder it is interesting to see the effect of exogenous GAGs. In these experiments, cultures were washed twice after GAG treatment and returned to culture in fresh exogenous GAG free, basal medium to minimize exogenous GAG contamination of later measurements. (Rooney et al., 2015). In this current study under basal conditions iAluRilⓇ significantly increased amounts of sGAG relative to control, increasing measured sGAGs by a factor of 15. After pre-treatment with PS, H_2_O_2_ or TNFα there was no significant effect of GAG treatment on sGAG production relative to control. Under TNFα conditions the amount of sGAG measured in the medium was increased but significantly greater only in the presence of iAluRilⓇ. A potential mode of action for GAG treatment in IC/BPS could be to promote endogenous GAG production in the urothelium thereby promoting urothelial regeneration and improved barrier function. Here we show that while all GAGs slight increase sGAG only iAluRilⓇ is effective at increasing sGAG production under inflammatory conditions.

The effect of GAG treatment on the levels of inflammatory IL-6 and IL-8 cytokines was next investigated. Under basal conditions, no GAG treatment had a significant effect on IL-6, while all GAG treatments were anti-inflammatory reducing IL-8 production over the 24 h treatment period which is consistent with previously published data (Rooney et al., 2015). Under inflamed conditions with TNFα pre-treatment none of the commercial GAG formulations significantly reduced IL-6 or IL-8 (except at 6 h for iAiluRil) production over a time course of 3–24 h. The literature suggests that lower molecular weight GAGs may result in favourable penetration into underlying tissues and receptor activation, and thus reducing mast cell activation and reduce pro-inflammatory cytokines like IL-6 and IL-8 (Vitanzo and Sennett, 2006). While higher molecular weights similar to those in the commercial GAGs used in this study may directly target urothelial barrier function and facilitate endogenous anti-inflammatory processes to take hold (Kallestrup et al., 2005). The lack of effect observed in this study compared to some previous work may reflect differences in the molecular weight of the GAG formulations used and if the formulation contained HA alone (CystistatⓇ and HyacystⓇ) or in combination with other GAGs like chondroitin sulfate (iAluRilⓇ).

Finally, the effect of GAG treatments on the expression of a range of genes was investigated.

There was no consistent trend in the expression of HA receptor genes and no GAG treatment had a significant effect on CD44 or HMMR gene expression. Only CystistatⓇ significantly increased ICAM-1 expression and this was only evident at 3-h under inflamed conditions, but returned to basal levels at 24 h. Urothelial damage in patients and animal models of IC/BPS has been shown to be associated with infiltration of activated mast cells and enrichment of the inflammatory milieu (Theoharides and O’Leary, 2006; Sant et al., 2007). While ICAM-1 is a HA receptor, it also plays a role in the inflammatory process. Intermediate molecular weight HA has been shown to increase the expression of ICAM-1 via the transcription factors NF-κB and AP-1 in kidney tubular cells (Oertli et al., 1998). A reduction in ICAM-1 is one of the potential routes through which HA alters IL-6 expression which correlates well with symptom severity in chemical induced cystitis models (Sahiner et al., 2018). Thus, the differential effects of the GAG formulation may reflect differences in molecular weight species in the various formulation. It is established that a compromised urothelium facilitates migration of toxic urinary solutes and electrolytes such as potassium, into the underlying layers of the bladder wall. Data from the literature supports candidates such as the urothelial cells and their associated GAG layer (Eldrup et al., 1983) which is found on the apical domain of the urothelial barrier. This is supported by others that have shown that a damage to the GAG layer leads to a loss of barrier function (Hurst et al., 2016). Previous data from our group has shown that a non-commercial HA formulation can have a significant positive effect on monolayer permeability (Rooney et al., 2015). Here however no GAG treatment had a significant effect on the expression of TJP-1 (Hurst et al., 2016). It should be noted that while we and others have used HTB-4 cells to model the bladder urothelium (Argade et al., 2016; Parsons et al., 2014) *in vitro* these cells are not a good model for barrier function assays, thus no change in tight junction proteins could be considered consistent with the cell model used in this study.

While there is some evidence here that both HyacystⓇ and iAluRilⓇ increased sGAG production measured in cell media, at the gene level there is no effect of GAG treatment on GAG synthesis enzymes CSGALNACT1 or CSGALNACT2. While the expression of CSGALNACT2 under both basal and inflamed conditions trended toward increasing over time there is a high degree of variability in the signal. While iAluRilⓇ in particular increased sGAG production as measured by DMMB assay, it appears that this is not due to increased expression of these two genes related to GAG synthesis.

Restoration of the urothelial layer is an important part of restoring barrier function, thus we examined the expression the of two genes associated with tissue remodeling. The extracellular matrix and GAG layer play a role in bladder barrier function the matrix-metalloproteinases (MMPs), have key role in the maintenance of the local environment. A unique function of MMP-2 is the ability to ability to degrade type IV collagen (Di Carlo et al., 2007). The management of MMP activity is highly regulated including functional inhibition by the tissue inhibitor of metalloproteinases (TIMPs). Studies suggest a role for supressed MMP2 production as a mechanism whereby IC/BPS cells have reduced cell proliferative and regeneration capacity (Keay and Zhang, 2016). However, in our study, we do not see any no change in the expression of TIMP1 or MMP2 under any condition with GAG treatment suggesting that these pathways are not target for GAG replacement therapies.

As pain is a central part of IC/BPS, we examined the expression of the genes for COX-induced prostaglandins and nerve growth factor (NGF). Both HyacystⓇ and iAluRilⓇ treatment of cells under basal conditions resulted in higher expression of COX-1/PTGS at 3 h compared to control. In inflamed cells, both HyacystⓇ and iAluRilⓇ reduced COX-1/PTGS expression at 3-h, there was no effect on COX-1/PTGS expression at 24 h. This differential effect on inflamed and non-inflamed cells contributes to the contradictory patient reported data previously (Engelhardt et al., 2011; Hanno et al., 2011). This highlight a potential anti-inflammatory, anti-nociceptive effect of HyacystⓇ and iAluRilⓇ while there was no effect of GAG treatment on NGF expression. Here we show that in an *in vitro* model of IC/BPS, intravesical GAG formulations are not deleterious to cell viability but do not greatly enhance wound healing. In addition, the GAG preparations while not effecting IL-6 production they suppress IL-8 production under basal conditions.

### Limitations

This *in vitro* experimental design while not reflecting the complexinty of the *in vivo* condition it was based on correlating IC/BPS pathogenesis and GAG-related pathways. The HTB-4 cells are a human urinary bladder transitional cell carcinoma cell line and as such are transformed and do not form tight monolayers similar to normal urothelium. In addition, it should be noted that the high levels of sGAG measured here in the presence of iAluRilⓇ could be due to the fact that sGAGs are a significant component of the iAluRilⓇ formulation itself, as it is difficult to distinguish exogenous and endogenous sGAGs when using iAluRilⓇ.

## Conclusions

The use of GAG treatments is popular with clinicians and patients with IC/BPS, due to factors such as the cost-effective nature of the treatments, and localized administration by the intravesical route. However, there is significant contradictory data about the mechanism of action of exogenous GAGs exists, as well as gaps in the understanding of the pathology of IC/BPS. This study aimed to examine the effects GAG treatments in IC/BPS in order to better understand the disease, and to identify therapeutic targets. The study has showed that intravesical preparations have moderate anti-inflammatory effects *in vitro* in response to disruption of the GAG layer and only minimal anti-inflammatory effects in the presence of the proinflammatory cytokine TNFα. Further research is required to elucidate more fully the mechanistic effects of exogenous GAGs in IC/BPS treatment, and ultimately to optimize treatment.

## Data Availability Statement

The original contributions presented in the study are included in the article/supplementary material, further inquiries can be directed to the corresponding author.

## Author Contributions

PR, AP, and LQ designed the studies. PR, CR, BM, and KD carried out the work. All author contributed to writing the manuscript.

## Funding

This publication has emanated from research conducted with the financial support of Science Foundation Ireland (SFI) and is co-funded under the European Regional Development Fund under Grant Number 13/RC/2073.

## Conflict of Interest

The authors declare that the research was conducted in the absence of any commercial or financial relationships that could be constructed as a potential conflict of interest.
